# Great Wall: A Generalized Dose Optimization Design for Drug Combination Trials Maximizing Survival Benefit

**DOI:** 10.1002/pst.70049

**Published:** 2025-11-12

**Authors:** Yan Han, Yingjie Qiu, Yi Zhao, Isabella Wan, Lang Li, Suyu Liu, Yong Zang

**Affiliations:** ^1^ Department of Biostatistics and Health Data Science Indiana University School of Medicine Indianapolis Indiana USA; ^2^ Peter O' Donnell Jr. School of Public Health University of Texas Southwestern Medical Center Dallas Texas USA; ^3^ Faculty of Arts and Science University of Toronto Toronto Ontario Canada; ^4^ Department of Biomedical Informatics The Ohio State University Columbus Ohio USA; ^5^ Department of Biostatistics The University of Texas MD Anderson Cancer Center Houston Texas USA; ^6^ Center for Computational Biology and Bioinformatics Indiana University School of Medicine Indianapolis Indiana USA

**Keywords:** dose optimization design, dose randomization, drug‐combination trial, immunotherapy, phase I–II clinical trial, survival outcome

## Abstract

Most phase I–II drug‐combination trial designs assume that selecting the optimal dose combination based on early outcomes will also lead to maximum long‐term survival benefits. However, this assumption is often violated in many clinical studies, generally due to high rates of relapse following the initial response. To address this problem, we propose the Great Wall design, a general dose optimization design for drug‐combination trials. The Great Wall design employs a “divide‐and‐conquer” algorithm to address the issue of partial order of toxicity and uses early outcomes to eliminate dose combinations that are excessively toxic or less efficacious. It utilizes a dose randomization approach to construct a candidate set of the promising dose combinations balancing the toxicity and early efficacy outcomes. The patients assigned to the candidate set are followed to collect the survival outcomes and the final optimal dose combination is then selected to maximize the survival benefit. The simulation studies confirm the desirable operating characteristics of the Great Wall design under various clinical settings. R codes are also provided to facilitate the application. The Great Wall design is modular and practically useful in settings where investigators plan to follow patients long enough to assess survival outcomes.

## 1 Introduction

The drug combination strategy, which involves administering two or more drugs together, has become popular due to its enhanced effectiveness and ability to delay drug resistance [1, 2, 3]. A notable example is the HER2‐positive breast cancer trial [4], where combining trastuzumab with chemotherapy significantly increased survival rates compared to chemotherapy alone.

A unique feature of the drug combination trials is that the toxicity order among dose combinations is only partially clear due to unknown drug–drug interaction effects, making the drug combination trial design more challenging than the single‐agent trial design [5]. Still, many phase I dose‐finding designs for drug combination trials have been proposed, which aim to identify the maximum tolerated dose (MTD) or MTD contour (e.g., multiple MTDs) based on the toxicity outcomes [6, 7, 8, 9, 10, 11, 12, 13].

Molecularly targeted agents (MTAs) and immunotherapies (ITs) have revolutionized cancer treatment. Unlike cytotoxic agents, the principle of “more is better” does not always apply to MTAs and ITs. Increasing their dose beyond the optimal therapeutic range may not enhance outcomes and could increase toxicity. Therefore, phase I–II trials for these agents focus on identifying the optimal biological dose (OBD), which balances toxicity and efficacy [14, 15]. Various dose optimization designs exist for drug combination trials to determine the OBD combination (ODC) [16, 17, 18, 19, 20, 21, 22, 23, 24].

In most phase I–II trials, early efficacy endpoints, like objective tumor response rate, are used to determine the OBD, followed by a phase III trial to confirm survival benefit. This approach aims to expedite dose optimization but has some very undesirable drawbacks. Firstly, small early‐phase sample sizes can lead to an OBD that proves unsafe or ineffective in sequential trials. Moreover, if short‐term efficacy is not a good surrogate for survival, there is a high risk of selecting a suboptimal dose for survival outcomes [25].

Identifying the OBD based solely on early outcomes without considering survival carries risks, as shown by a phase II trial comparing busulfan plus melphalan (B+M) with melphalan (M) alone for multiple myeloma. The trial was terminated early due to lower response rates for B+M (13.6%) versus M (40.6%). However, post hoc analysis revealed 12‐month PFS probabilities of 0.90 for B+M versus 0.77 for M, leading to a redesign with PFS as the primary outcome [26]. This highlights that early efficacy may not always correlate with survival. Recent hybrid designs [27, 28, 29] integrate early and long‐term outcomes, but none are tailored for drug–drug combination trials.

In 2021, FDA initiated Project Optimus [30] which aims to improve oncology drug development by shifting focus from the traditional maximum tolerated dose (MTD) based approach to more evidence‐based dosing strategies. A key component of this initiative is the emphasis on dose randomization during clinical trials. Rather than testing only the highest tolerated dose, dose randomization involves evaluating multiple dose levels across trial participants. This allows for a more comprehensive understanding of the drug's efficacy and safety profile at different doses and higher accuracy in identifying the true optimal dose.

In this paper, we propose the Great Wall design, a new dose optimization design for drug–drug combination trials that integrates dose escalation and adaptive dose randomization to identify the ODC maximizing survival benefit. The design consists of three stages. In stage 1, a “divide‐and‐conquer” dose escalation algorithm rapidly explores the toxicity and early efficacy profiles, establishing a set of admissible dose combinations based on early outcomes. Stage 2 randomizes more patients to the admissible set and refines it using a mean utility criterion that balances toxicity and efficacy to construct the candidate set. In stage 3, patients are further randomized to the candidate set, with survival outcomes used to select the final ODC at the end of the trial.

## 2 Adaptive Design

Consider a drug combination trial with two drugs, A and B, each with multiple doses, $A_{1}<\cdots <A_{J}$, and $B_{1}<\cdots <B_{K}$, respectively. Without loss of generality, the doses of drug A are arranged in descending order vertically, while the doses of drug B are arranged in ascending order horizontally. The dose combinations are represented in a J×K matrix, where the optimal dose combination (ODC) maximizes survival benefit while maintaining acceptable toxicity. We propose the Great Wall design, a three‐stage approach that identifies the ODC by evaluating toxicity (e.g., DLT), early efficacy (e.g., tumor response), and survival outcomes.

Stage 1 involves a rapid toxicity assessment to eliminate dose combinations with unacceptable toxicity. However, the toxicities are monotonically increasing among the dosage of one drug only if the dosage for the other drug is fixed, known as the partial toxicity order. To address this issue, we employ a “divide and conquer” algorithm that divides the dose matrix into sub‐paths, ensuring ordered toxicity probabilities within each, allowing for the use of single‐agent dose‐finding methods.

Starting with the original dose matrix $M_{1}$, the first sub‐path $S_{1}$ includes the dose combinations in the leftmost column and top row, arranged in increasing toxicity as:

$$A_{1}B_{1}<\cdots <A_{J}B_{1}<A_{J}B_{2}<\cdots <A_{J}B_{K}.$$

Unlike the conventional dose‐finding designs identifying MTD, dose escalation following this sequence aims to identify combinations with excessive toxicity, without the need to retain doses. Therefore, the dose escalation process continues as long as the current dose remains safe.

Let $l_{d}$ represent the number of patients treated at current dose combination d, and $x_{T,d}$ be the number who experienced DLTs. The toxicity probability estimate is ${\hat{p}}_{T,d}=\frac{x_{T,d}\;}{l_{d}}$, and ψ denotes the threshold for excessive toxicity. If ${\hat{p}}_{T,d}<\psi$, we continue dose escalation within $S_{1}$. If ${\hat{p}}_{T,d}\geq \psi$, escalation stops, and the current dose combination and all the combinations beyond are deemed overly toxic.

We propose a model‐assisted design framework to optimize ψ. Let $\emptyset_{T}$ be the well‐tolerated toxicity probability, and $\rho \emptyset_{T}$ be the toxicity rate that is deemed overly toxic (e.g., ρ=1.4 following the BOIN design). We formulate two simple hypotheses at current dose combination as:

$$H_{0}:p_{T,d}=\emptyset_{T}⟷H_{1}:p_{T,d}=\rho \emptyset_{T}.$$

So, if $H_{0}$ is true, the correct decision is to keep the dose escalation, which would happen only if ${\hat{p}}_{T,d}<\psi$ is observed. Similarly, if $H_{1}$ is true, the correct decision is to terminate the dose escalation with ${\hat{p}}_{T,d}\geq \psi$ being observed. Finally, the overall correct decision probability is:

(1) $$P\left(\psi \right)=\mathrm{Pr}\left(H_{0}\right)*\mathrm{Pr}\left(\;{\hat{p}}_{T,d}<\psi |H_{0}\right)+\mathrm{Pr}\left(H_{1}\right)*\mathrm{Pr}\left(\;{\hat{p}}_{T,d}\geq \psi |H_{1}\right).$$

where

$$\mathrm{Pr}\left(\;{\hat{p}}_{T,d}<\psi |H_{0}\right)=\mathrm{Bin}\left(l_{d}\psi -1;l_{d},\emptyset_{T}\right)$$

$$\mathrm{Pr}\left(\;{\hat{p}}_{T,d}\geq \psi |H_{1}\right)=1-\mathrm{Bin}\left(l_{d}\psi -1;l_{d},\rho \emptyset_{T}\right).$$

Let $b_{d}=\text{floor}\left(l_{d}\psi -1\right)$ and assign equal priors $\mathrm{Pr}\left(H_{0}\right)$ and $\mathrm{Pr}\left(H_{1}\right)$, $P\left(\psi \right)$ can be expressed as

Pψ=12Binldψ−1ld∅T+121−Binldψ−1ldρ∅T=12∑xT,d=0bdldxT,d∅TxT,d1−∅Tld−xT,d−ρ∅TxT,d1−ρ∅Tld−xT,d+12=∑xT,d=0bd12ldxT,dρ∅TxT,d1−ρ∅Tld−xT,d∅Tρ∅TxT,d1−∅T1−ρ∅Tld−xT,d−1+12.

Because ρ>1, ${\left(\frac{\emptyset_{T}}{\rho \emptyset_{T}}\right)}^{x_{T,d}}{\left(\frac{1-\emptyset_{T}}{1-\rho \emptyset_{T}}\right)}^{l_{d}-x_{T,d}}$ monotonically decreasing with $x_{T,d}$. Therefore, given 12ldxT,dρ∅TxT,d1−ρ∅Tld−xT,d>0, $P\left(\psi \right)$ is maximized when

$$l_{d}\psi =\max\left\{x_{T,d}:{\left(\frac{\emptyset_{T}}{\rho \emptyset_{T}}\right)}^{x_{T,d}}{\left(\frac{1-\emptyset_{T}}{1-\rho \emptyset_{T}}\right)}^{l_{d}-x_{T,d}}\geq 1\right\}.$$

This leads to optimal value of ψ:

$$\psi =\frac{\log\left(\frac{1-\emptyset_{T}}{1-\rho \emptyset_{T}}\right)}{\log\left(\rho \left(\frac{1-\emptyset_{T}}{1-\rho \emptyset_{T}}\right)\right)}.$$

A key feature of the “divide and conquer” algorithm is its ability to reduce the searching space for subsequent sub‐paths automatically. For instance, if during dose escalation in sub‐path $S_{1}$, the dose combination $A_{j}B_{k}$ is deemed overly toxic, then any combination $A_{j^{*}}B_{k^{*}}$ with $j^{*}>j$ or $k^{*}>k$ is also overly toxic and excluded. Let $E_{1}$ be these excluded combinations. After $S_{1}$, the search space reduces to $M_{2}=M_{1}\backslash \left(S_{1}\cup E_{1}\right)$, and this process is repeated for all sub‐paths.

We acknowledge that there are multiple ways to configure sub‐paths, and the configuration used in this paper is for illustration only. In practice, physicians can tailor configurations based on clinical and pharmacometric knowledge of toxicity profiles. For example, if increasing drug B's dosage potentially induces more overlapping toxicity than drug A, sub‐paths can prioritize escalating drug A to enhance safety. Nevertheless, the proposed dose escalation design is flexible and applicable to any reasonable configuration.

**FIGURE 1:**
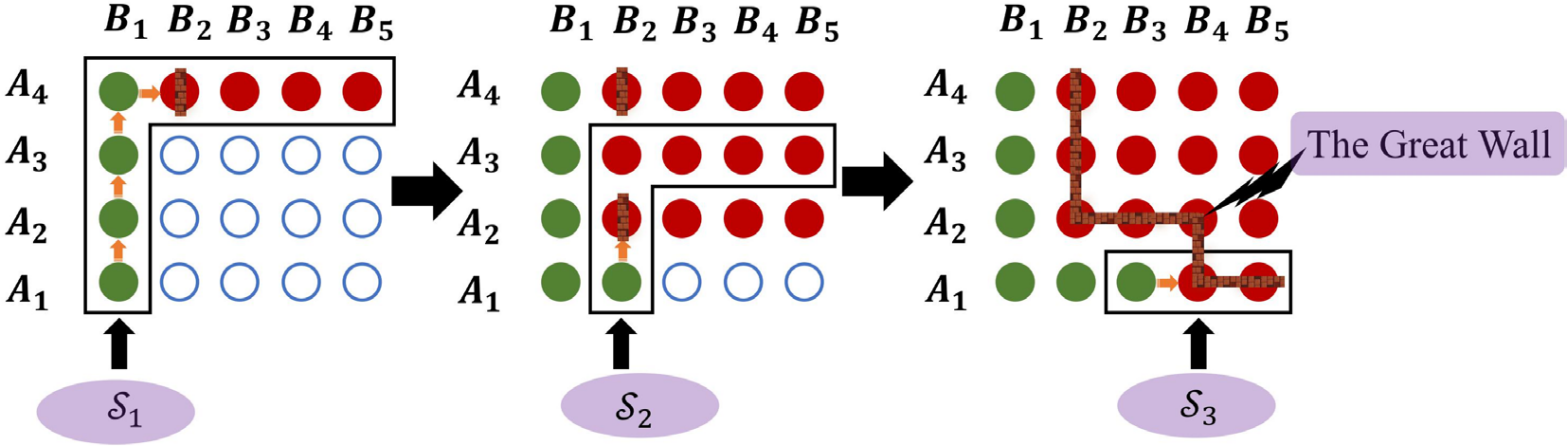
An illustrative example for stage 1 of the Great Wall design.

Figure 1 illustrates stage 1 of the Great Wall design in a 4 × 5 drug combination trial. Green represents safe doses, red denotes unsafe ones, and hollow circles indicate untried doses. The first sub‐path is $S_{1}=\left\{A_{1}B_{1},A_{2}B_{1},A_{3}B_{1},A_{4}B_{1},A_{4}B_{2},A_{4}B_{3},A_{4}B_{4},A_{4}B_{5}\right\}$. We start at $A_{1}B_{1}$ and escalate through $S_{1}$ until $A_{4}B_{2}$ is identified as overly toxic and build the first “wall” there. The searching space reduces to $M_{2}=M_{1}\backslash S_{1}$ and $S_{2}=\left\{A_{1}B_{2},A_{2}B_{2},A_{3}B_{2},A_{3}B_{3},A_{3}B_{4},A_{3}B_{5}\right\}$. By escalating through $S_{2}$ we identify $A_{2}B_{2}$ as overly toxic, forming the second wall and further reducing space. The final search space is $S_{3}=\left\{A_{1}B_{3},A_{1}B_{4},A_{1}B_{5}\right\}$. After building the third wall at $A_{1}B_{4}$, “the Great Wall” can be constituted by linking all the walls together. Then, all the doses below the “the Great Wall” are safe and can be moved to the next stage.

Stage 1 primarily focuses on toxicity outcomes for dose escalation, but early efficacy and survival outcomes can also be collected if the trial's eligibility criteria remain consistent across stages. This seamless data integration enhances the identification of the ODC. However, if no major efficacy benefit is expected for patients enrolled in stage 1, only toxicity outcomes should be collected. For this paper, we assume population homogeneity and collect all outcomes starting from stage 1.

Let $A_{T,1}$ be the admissible set of toxicity for stage 1, including all the dose combinations below the Great Wall. This set is further refined by eliminating dose combinations with ineffective early efficacy profiles. For each $d\in A_{T,1}$, we assign a Beta prior distribution $\text{Beta}\left(1,1\right)$ for the early efficacy probability $p_{E,d}$. Based on the observed data $D_{1}$ in stage 1, we can derive the corresponding posterior distribution $\left[p_{E,d}|D_{1}\right]$, also from a Beta distribution. Then, let $\emptyset_{E}$ be the fixed lower limit for the early efficacy endpoint, the admissible set for stage 1, including dose combinations with acceptable toxicity and early efficacy profiles, is:

(2) $$A_{1}=\left\{d\in A_{T,1}:\mathrm{Pr}\left(p_{E,d}>\emptyset_{E}|D_{1}\;\right)>0.05\right\}.$$

We use 0.05 as the cut‐off for unacceptable early efficacy, with 0.05 to 0.2 generally working well in preliminary simulations. Due to the limited sample size used in stage 1, the purpose of developing $A_{1}$ is to exclude the dose combination that are extremely toxic or inefficacious rather than providing a comprehensive toxicity and efficacy evaluation. A small cut‐off value provides such flexibility and has been widely adopted in various dose optimization designs [14, 27, 28, 29]. The value can also be determined by clinicians to reflect different clinical requirements.

In stage 1, the inability to retain doses limits us to assigning only one cohort per dose combination, impacting the accuracy of our estimates. In stage 2, we equally randomize additional $n_{2}$ patients among the admissible set $A_{1}$ and gather data $D_{2}$. Although toxicity profiles were assessed in stage 1, the primary objective is to exclude overly toxic dose combinations rather than to pinpoint the MTD contours. In clinical practice, it is generally infeasible to select an ODC beyond the MTD contour. Therefore, by the end of stage 2, we aim to enhance trial safety by identifying the MTD contour.

We begin by estimating toxicity probabilities for all the tested dose combinations based on the combined data $D_{1}\cup D_{2}$, using matrix isotonic regression [10] to preserve partial toxic ordering. Then, for each row of the dose matrix, we select the MTD as the dose combination with a toxicity estimate closest to the well‐tolerated toxicity rate $\emptyset_{T}$, provided no combination is excessively toxic. The dose combinations at or below the MTD contour constitute the stage 2 toxicity admissible set $A_{T,2}$. After updating the posterior distribution $\left[p_{E,d}|D_{1}\cup D_{2}\right]$, the stage 2 overall admissible set is:

(3) $$A_{2}=\left\{d\in A_{T,2}:\mathrm{Pr}\left(p_{E,d}>\emptyset_{E}|D_{1}\cup D_{2}\;\right)>0.05\right\}.$$

Since $A_{2}$ may include many dose combinations, we streamline it by selecting the most promising candidates using the mean utility function, which balances toxicity and early efficacy.

Let $Y_{T}$ represent the binary toxicity outcome ($Y_{T}=1$ for DLT, $Y_{T}=0$ otherwise) and $Y_{E}$ represent early efficacy ($Y_{E}=1$ for complete or partial remission, $Y_{E}=0$ otherwise). We define $\pi_{a,b}\left(d\right)=\mathrm{Pr}\left(Y_{E}=a,Y_{T}=b|d\right)$ as the joint probability for $\left(Y_{E},Y_{T}\right)$ at dose combination d, where $a,b\in \left\{0,1\right\}$. To establish utility, we assign $U_{1,0}=100$ to the most desirable outcome $\left(Y_{E}=1,Y_{T}=0\right)$, and $U_{0,1}=0$ to the least desirable $\left(Y_{E}=0,Y_{T}=1\right)$. Clinicians can then provide subjective utility scores for $\left(Y_{E}=1,Y_{T}=1\right)$ and $\left(Y_{E}=0,Y_{T}=0\right)$ between 0 and 100.

The mean utility of dose d, normalized between 0 and 1, is $\bar{U}\left(d\right)=\frac{1}{100}\sum_{a=0}^{1}\sum_{b=0}^{1}U_{a,b}\pi_{a,b}\left(d\right)$.

This approach can handle ordinal endpoints and is easily interpretable for clinicians, making it widely used in early‐phase trial designs [14].

Let $l_{d}$ be the number of patients treated at the dose combination d and $x_{a,b,d}$ of them have experienced the event $\left(Y_{E}=a,Y_{T}=b\right)$. At the end of stage 2, for each dose combination $d\in A_{2}$, by plugging in the simple proportional estimates $\hat{\pi_{a,b}}\left(d\right)=\frac{x_{a,b,d}}{l_{d}\;}$, we calculate $\hat{U}\left(d|D_{1}\cup D_{2}\right)$ as the estimate of the mean utility at dose combination d using the accumulated data $D_{1}\cup D_{2}$, and identify ${\hat{U}}^{\max}=\max\left\{\hat{U}\left(d|D_{1}\cup D_{2}\right),d\in A_{2}\right\}$. Then, we can construct the candidate dose set for stage 2 as:

(4) $$C_{2}=\left\{d\in A_{2}:\hat{U}\left(d|D_{1}\cup D_{2}\right)\geq \gamma {\hat{U}}^{\max}\right\}.$$

γ in (4) determines the flexibility of candidate set selection. For example, if γ=70%, then any dose combination $d\in A_{2}$ with estimated mean utility at least 70% of the maximum value is included in the candidate set.

In stage 3, after establishing $C_{2}$, we randomize the final cohort of $n_{3}$ patients equally within $C_{2}$. We then complete follow‐ups for all patients assigned to $C_{2}$ throughout the trial, collecting survival and toxicity data. Using the cumulative toxicity data, we construct the final MTD contour and update the candidate dose set to $C_{3}$, excluding doses above the contour to further strengthen the safety. Let $\psi_{S}\left(d\right)$ represent the dose‐optimality criterion (e.g., survival rate, median survival), with $\hat{\psi_{S}}\left(d|D_{1}\cup D_{2}\cup D_{3}\right)$ denoting the Kaplan–Meier estimator using all survival data from the trial. The final optimal dose combination (ODC) is selected as:

$${\hat{d}}^{\mathrm{opt}}={\text{argmax}}_{d\in C_{3}}\hat{\psi_{S}}\left(d|D_{1}\cup D_{2}\cup D_{3}\right).$$

**FIGURE 2:**
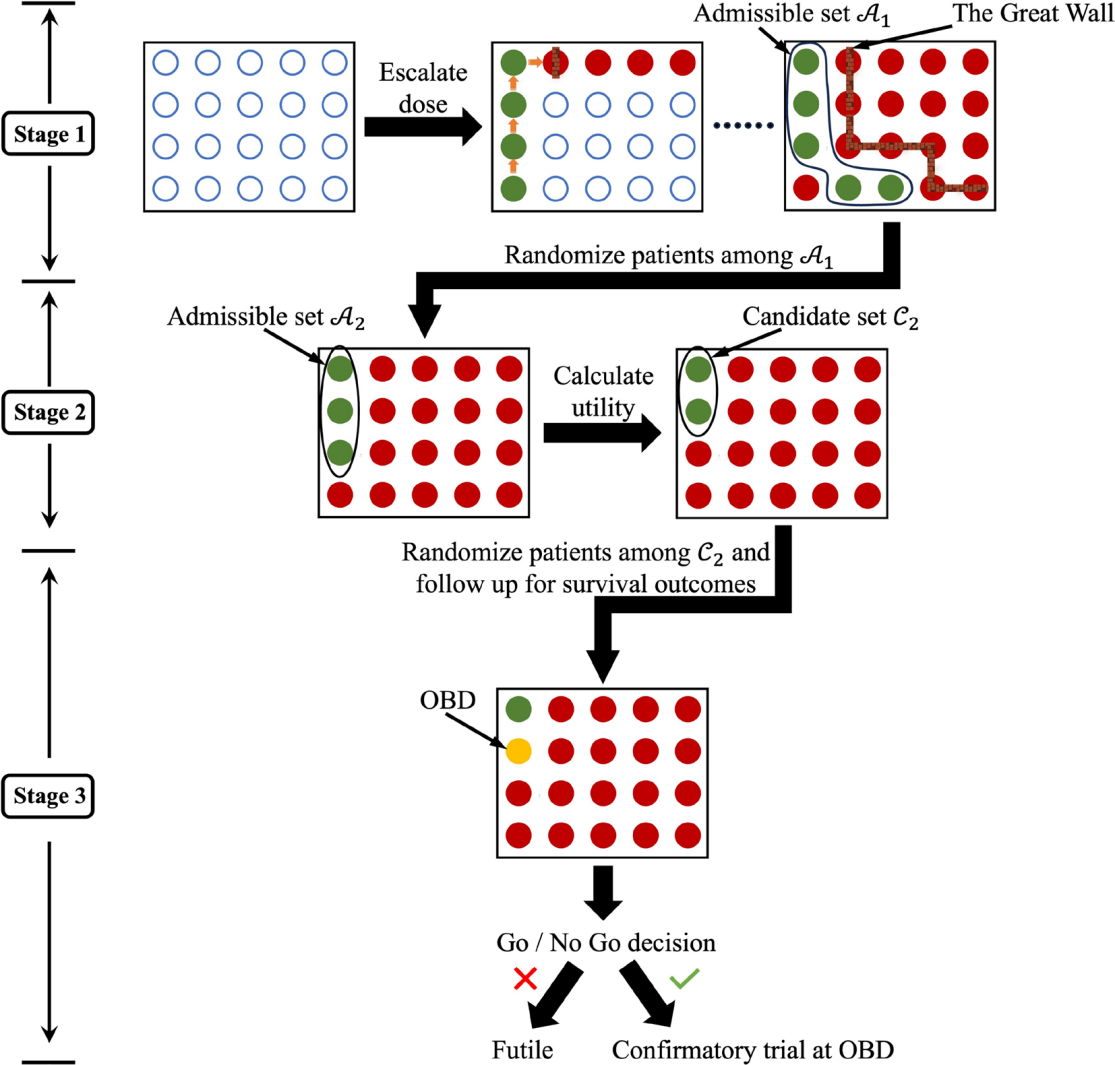
Schematic for the Great Wall design.

Finally, a Go/No‐Go decision is made. Let $\emptyset_{S}$ be the lower limit for the survival metric. If $\hat{\psi_{S}}\left({\hat{d}}^{\mathrm{opt}}|D_{1}\cup D_{2}\cup D_{3}\right)>\emptyset_{S}$, the decision is “Go,” and a large‐scale randomized trial should follow. Otherwise, a “No Go” decision is made, deeming the trial futile. A schematic for the proposed Great Wall design is provided in Figure 2.

## 3 Simulation Studies

We conducted simulation studies to assess the performance of the Great Wall design across eight scenarios varying in true toxicity, early efficacy, and survival probabilities (Table 1). To our knowledge, no dose optimization design for drug‐combination trials currently considers survival outcomes. Thus, we compared the Great Wall design with two utility‐based phase I–II designs: CONV1 and CONV2. The CONV1 design uses stages 1 and 2 of the Great Wall design but excludes survival data, selecting the optimal dose combination (ODC) by maximizing mean utility at the end of stage 2. The CONV2 design first identifies the MTD contour using the waterfall design [10], then randomizes patients across the MTD contour, collects survival outcomes, and selects the ODC based on survival data. Sample sizes for the CONV1 and CONV2 designs were matched to those of the Great Wall design for a fair comparison.

**TABLE 1:** True toxicity, early efficacy, mean utility, and PFS probability $\left(\begin{array}{cc} p_{T,d} & p_{E,d}\\ \bar{U}\left(d\right) & \psi_{S}\left(d\right) \end{array}\right)$ of Scenarios 1 to 8 for simulation studies.

	Drug B			Drug B		
	1	2	3	1	2	3
Drug A	Scenario 1			Scenario 2		
2	(0.60,0.20, 0.28,0.25)	(0.67,0.40, 0.37,0.50)	(0.75,0.30, 0.28,0.40)	(0.25,0.45, 0.57,0.10)	(0.40,0.55, 0.57,0.15)	(0.50,0.50, 0.50,0.20)
1	(0.5,0.10, 0.26,0.20)	(0.62,0.20, 0.27,0.40)	(0.68,0.30, 0.31,0.50)	(0.10,0.40, 0.60,0.05)	(0.35,0.50, 0.56,0.10)	(0.45,0.50, 0.52,0.25)
Drug A	Scenario 3			Scenario 4		
2	(0.20,0.53, 0.64,0.35)	(0.40,0.40, 0.48,0.25)	(0.50,0.40, 0.44,0.40)	(0.10,0.3, 0.54,0.35)	**(0.15,0.60, 0.70,0.65)**	(0.45,0.55, 0.55,0.40)
1	**(0.10,0.55, 0.69,0.55)**	(0.35,0.60, 0.62,0.35)	(0.45,0.60, 0.58,0.20)	(0.05,0.20, 0.50,0.20)	(0.10,0.40, 0.60,0.40)	(0.40,0.40, 0.48,0.45)
Drug A	Scenario 5			Scenario 6		
2	**(0.15,0.40, 0.58,0.45)**	(0.20,0.60, 0.68,0.25)	(0.45,0.45, 0.49,0.35)	(0.10,0.60, 0.72,0.42)	(0.25,0.60, 0.66,0.42)	(0.48,0.65, 0.60,0.30)
1	(0.10,0.20, 0.48,0.20)	(0.15,0.30, 0.52,0.25)	(0.40,0.30, 0.42,0.40)	(0.08,0.30, 0.55,0.25)	**(0.18,0.55, 0.66,0.60)**	(0.42,0.55, 0.56,0.35)
Drug A	Scenario 7			Scenario 8		
2	(0.12,0.20, 0.47,0.20)	(0.15,0.58, 0.69,0.40)	(0.17,0.62, 0.70,0.40)	(0.06,0.20, 0.50,0.25)	(0.10,0.40, 0.60,0.40)	**(0.12,0.70, 0.77,0.65)**
1	(0.08,0.14, 0.45,0.20)	(0.12,0.30, 0.53,0.25)	**(0.14,0.60, 0.70,0.65)**	(0.02,0.10, 0.45,0.20)	(0.05,0.20, 0.50,0.35)	**(0.10,0.28, 0.53,0.65)**

*Note:* True ODCs are gray highlighted in boldface. $\psi_{S}\left(d\right)$ indicates 6‐months PFS rate.

Based on clinician input and the clinical trial practice, we decide the following values for the simulation study. For the admissible set $A_{1}$ in stage 1, we set $\emptyset_{T}=0.3$ and $\emptyset_{E}=0.25$. For the mean utility $\bar{U}\left(d\right)$, we set $U_{0,0}=40$, $U_{0,1}=0$, $U_{1,0}=100$, and $U_{1,1}=60$. The PFS assessment window was 6 months, and the dose‐optimality criterion $\psi_{S}\left(d\right)$ is defined as the 6‐months PFS rate with a Go/No Go decision cut‐off of $\emptyset_{S}=0.3$. For the candidate dose set $C_{2}$ in stage 2, we set γ=70% based on simulation studies (see sensitivity analysis). The joint toxicity‐early efficacy outcome was generated using the Gumbel model [31], with an association parameter of 0.5. Survival data followed a Weibull distribution. Patients in stage 1 were treated in cohorts of size 3, with maximum sample sizes $n_{1}=18$, $n_{2}=36$, and $n_{3}=20$, determined from preliminary simulation studies.

Table 2 summarizes the operating characteristics of the Great Wall and CONV designs, including the ODC selection percentages, percentages of patients treated at each dose combination, and mean overall sample size. The “No Sel %” column represents the percentage of trials concluding that no dose combination should be selected by the end of the trial. All results are based on 10,000 simulated replicates of the trial using each design.

**TABLE 2:** Simulation results of the Great Wall, CONV1, and CONV2 designs.

Designs	Sel %			No Sel %	Pat %			Sample size
Scenario 1								
Great Wall	0.0	0.0	0.0	**99.4**	14.3	3.2	0.5	32.2
	0.6	0.0	0.0		65.3	13.7	3.0	
CONV1	0.0	0.0	0.0	**88.9**	15.6	3.6	0.6	39.2
	11.1	0.0	0.0		63.6	13.9	3.0	
CONV2	2.1	0.1	0.0	**96.3**	11.1	0.4	0.0	33.2
	1.1	0.4	0.0		87.1	1.3	0.0	
Scenario 2								
Great Wall	0.2	0.3	0.0	**98.0**	23.5	10.9	5.1	72.0
	0.0	0.3	1.2		32.6	19.3	8.6	
CONV1	21.2	4.1	0.2	**2.9**	22.4	13.4	6.6	72.0
	48.3	19.5	3.9		29.0	18.5	10.1	
CONV2	0.0	0.1	7.4	**91.9**	28.2	14.8	3.8	73.5
	0.1	0.4	0.1		18.5	22.7	12.1	
Scenario 3								
Great Wall	5.6	0.1	0.0	3.1	25.1	11.2	5.3	71.7
	**86.3**	4.9	0.0		31.3	18.7	8.4	
CONV1	18.3	0.2	0.0	3.0	23.9	13.9	6.6	72.0
	**61.0**	15.2	2.4		28.7	17.6	9.3	
CONV2	19.7	7.4	4.8	18.3	26.2	17.8	4.9	73.6
	**21.4**	26.8	1.6		17.1	21.4	12.7	
Scenario 4								
Great Wall	5.3	**77.1**	0.3	1.9	19.3	21.6	8.9	73.1
	0.3	11.1	4.0		18.0	20.6	11.6	
CONV1	5.9	**63.9**	1.7	0.9	19.9	17.8	10.7	73.0
	2.8	21.7	3.1		19.8	19.0	12.8	
CONV2	2.5	**58.5**	26.7	2.9	8.7	26.8	24.0	73.9
	0.0	3.0	6.4		5.8	12.3	22.5	
Scenario 5								
Great Wall	**57.4**	8.6	0.8	19.1	21.9	20.2	8.2	71.2
	1.7	6.3	6.0		19.5	19.3	11.0	
CONV1	**22.0**	55.4	0.9	2.7	19.9	16.8	9.9	72.1
	5.2	11.9	1.8		21.1	19.3	13.0	
CONV2	**15.5**	8.0	16.1	25.6	12.8	23.8	17.6	73.6
	0.0	6.1	28.7		10.0	15.1	20.7	
Scenario 6								
Great Wall	20.4	8.9	0.1	2.5	24.1	17.0	7.6	71.8
	1.2	**65.6**	1.2		19.6	20.7	11.0	
CONV1	52.6	15.3	0.8	1.9	21.2	17.0	9.4	72.7
	2.4	**23.3**	3.8		21.4	19.0	12.0	
CONV2	6.5	26.0	8.3	10.1	12.3	28.1	14.0	73.8
	0.7	**35.9**	12.5		7.9	16.8	20.8	
Scenario 7								
Great Wall	0.3	11.2	8.4	3.8	14.3	18.7	16.7	72.1
	0.1	0.7	**75.4**		14.6	16.1	19.5	
CONV1	0.3	27.1	28.9	1.6	18.0	16.6	14.2	72.6
	0.3	2.7	**39.1**		18.1	17.1	16.0	
CONV2	0.3	1.6	60.3	7.9	9.9	12.6	47.4	73.8
	0.0	0.8	**29.1**		7.1	2.2	20.8	
Scenario 8								
Great Wall	0.3	4.2	**71.4**	0.5	15.0	18.2	22.7	73.8
	0.0	1.3	**22.3**		13.7	15.0	15.5	
CONV1	1.3	8.2	**85.2**	0.1	17.8	17.2	15.2	73.9
	0.2	1.7	**3.4**		16.8	16.7	16.2	
CONV2	0.0	6.7	**80.3**	0.1	5.6	9.6	63.8	73.9
	0.0	0.0	**12.9**		4.4	0.3	16.3	

*Note:* Sel % is the percentage of selecting the dose combination as the ODC. No Sel % is the percentage of claiming no ODC existing. Pat % is the percentage of patients treated at the dose combination. Boldface and gray highlighter indicate values for the true ODC.

Scenarios 1 and 2 are null cases where no dose combination satisfies both toxicity and survival criteria. In scenario 1, all combinations are too toxic, while in scenario 2, although combinations $A_{1}B_{1}$ and $A_{2}B_{1}$ meet the safety criterion, they show unacceptable PFS rates. The Great Wall design correctly recommends no dose combination 99.4% of the time in scenario 1 and 98.0% in scenario 2. For the CONV1 design, the corresponding percentages are 88.9% in scenario 1 and only 2.9% in scenario 2, due to the absence of survival monitoring. The CONV2 design, which includes survival outcomes, yields 96.3% and 91.9% no‐selection percentages in scenarios 1 and 2, respectively.

In scenarios 3 and 4, the true ODC matches the dose combination with the highest mean utility. The Great Wall design outperforms CONV1, with correct ODC selection rates 25.3% and 13.2% higher in scenarios 3 and 4, respectively. CONV2 performs worst due to its focus on the MTD for the candidate set. In scenario 5, $A_{2}B_{1}$ is the true ODC with the highest survival rate of 0.45, while $A_{2}B_{2}$ has the highest mean utility of 0.68. The Great Wall design correctly selects $A_{2}B_{1}$ 57.4% of the time, compared to 22.0% and 15.5% for CONV1 and CONV2. Scenario 6 results are similar to scenario 5.

In scenario 7, three dose combinations, $A_{2}B_{2}$, $A_{1}B_{3}$, and $A_{2}B_{3}$ have similar mean utility around 0.7, but only $A_{1}B_{3}$ is the true ODC. The Great Wall design outperforms CONV1 and CONV2, improving correct ODC selection by 36.3% and 46.3%, respectively. In scenario 8, $A_{1}B_{3}$ and $A_{2}B_{3}$ are the true ODCs with the highest survival rate of 0.65. The Great Wall design correctly identifies them 93.7% of the time, compared to 88.6% for CONV1. CONV2 performs similarly, selecting the ODCs 93.2% of the time since $A_{1}B_{3}$ or $A_{2}B_{3}$ fall within the MTD contour. For patients' allocation, the Great Wall and CONV1 designs allocate more patients to efficacious combinations than the CONV2 design.

We conducted sensitivity analyses to assess the Great Wall design's performance across various parameters, with results provided in the Supporting Information. In the main simulations, patients were followed for 6 months to collect PFS outcomes and select the optimal dose. Figure S1 shows the results for different follow‐up times, confirming that longer follow‐up improves true ODC selection. However, the difference between 2‐ and 6‐month follow‐up is minimal, suggesting that shorter follow‐up is feasible, which enhances the practicality of the Great Wall design.

Figure S2 shows the results using different values of γ to construct the candidate dose set in formula (4). The results confirm that the original value of γ=0.7 reports the best overall performances across all the scenarios for simulation. Figure S3 depicts the results using different utility scores. We consider additional configurations of the utility scores as (1) $U_{0,0}=55$, $U_{0,1}=0$, $U_{1,0}=100$, $U_{1,1}=45$; and (2) $U_{0,0}=30$, $U_{0,1}=0$, $U_{1,0}=100$ and $U_{1,1}=100$. The configuration (3) is the original one used for the main simulation studies. The results show that Great Wall design is not sensitive to the choices of the utility scores.

Table S1 explores the impact of including stage 2 in the Great Wall design by comparing it to a modified version, Great Wall‐M, which combines stages 2 and 3. After identifying the admissible set $A_{1}$ in stage 1, the Great Wall‐M design skips stage 2 and directly randomizes patients within $A_{1}$, using the combined sample size originally allocated to stages 2 and 3. It then collects survival outcomes to select the optimal dose. Table S1 shows that the original Great Wall design provides a mild to moderate improvement in correctly identifying the true optimal dose, confirming the importance of stage 2. Table S2 presents the results for different stage 1 cohort sizes of 3 and 6. The findings confirm that the original cohort size of 3 provides more robust performance than a cohort size of 6. Table S3 compares the Great Wall design with a modified Waterfall design, which applies the Waterfall design [10] with the combined stage 1 and stage 2 sample size to identify the MTD contour before applying the same mean utility function and stage 3 procedure to select the ODC. The results demonstrate the superior performance of the Great Wall design.

Lastly, we also provide a trial illustration example in the Supporting Information, and the illustrative data are summarized in Tables S4 and S5.

## 4 Software

R code for implementing the Great Wall design is available from https://github.com/yongzang2020.

## 5 Discussion

In this paper, we propose a novel dose optimization design for drug‐combination trials referred to as the Great Wall design. This design addresses a critical limitation of conventional phase I/II methods, which often suffer from weak correlations between early efficacy outcomes and long‐term survival outcomes. The Great Wall design uses a “divide‐and‐conquer” algorithm to tackle the issue of partial order and uses the early outcomes to exclude the overly toxic and less efficacious dose combinations. A candidate set containing the most promising dose combinations is constructed through the mean utility method balancing the toxicity and early efficacy outcomes. The patients assigned to the candidate set are followed to collect the survival outcomes and the final ODC is then selected to maximize the survival benefit. Simulation studies confirm the desirable performance of the Great Wall design.

In clinical practice, it is possible that the admissible set of stage 1 $A_{1}$ contains many dose combinations whereas the planned stage 2 sample size $n_{2}$ is relatively small due to the feasibility of the trial. If that happens, a practical resolution is to further screen $A_{1}$ based on the toxicity‐efficacy tradeoff such that only a few most promising candidates can be selected for further evaluation.

The Great Wall design is modular. The early outcomes can be any ordinal outcomes used by a dose‐finding design. Any phase I design can be used to guide the dose escalation/de‐escalation within each sub‐path, with slight modification. As the objective of stage 1 is to exclude overly toxic dose combinations rather than identifying the MTD, a “dose retaining” decision from the conventional phase I design should be changed to “dose escalation,” and a “dose de‐escalation” decision should be changed to “sub‐path termination.” Any early and long‐term optimality criterion can be used for stages 2 and 3 to construct the candidate set and select the ODC, respectively. The mean utility method is general and contains the marginal toxicity and early efficacy distributions as special cases. Thus, the Great Wall design can be tailored to accommodate various clinical settings and is practically useful in settings where investigators plan to follow patients long enough to assess survival outcomes.

## Conflicts of Interest

The authors declare no conflicts of interest.

## Supporting information


**Figure S1:** Sensitivity analysis of the Great Wall design to different follow up times.
**Figure S2:** Sensitivity analysis of the Great Wall design to different values of γ in the candidate dose set.
**Figure S3:** Sensitivity analysis of the Great Wall design to different utility scores.
**Table S1:** Simulation results of the Great Wall and Great Wall‐M designs. Sel % is the percentage of selecting the dose combination as the ODC. No Sel % is the percentages of claiming no ODC existing. Pat % is the percentages of patients treated at the dose combination. Boldface and gray highlighter indicates values for the true ODC.
**Table S2:** Simulation results of the Great Wall designs under different cohort sizes in stage I. Great Wall‐3 and Great Wall‐6 use cohort sizes of 3 and 6 in stage I respectively. Sel % is the percentage of selecting the dose combination as the ODC. No Sel % is the percentages of claiming no ODC existing. Pat % is the percentages of patients treated at the dose combination. Boldface and gray highlighter indicates values for the true ODC.
**Table S3:** Simulation results of the Great Wall design and modified Waterfall design. Sel % is the percentage of selecting the dose combination as the ODC. No Sel % is the percentages of claiming no ODC existing. Pat % is the percentages of patients treated at the dose combination. Boldface and gray highlighter indicates values for the true ODC.
**Table S4:** Patient level data for toxicity and early efficacy outcomes for the trial example.
**Table S5:** Patient level data for survival outcomes (in days).

## Data Availability

Data sharing is not applicable to this article as no new data were created or analyzed in this study.
