# Supplementary material for: Great Wall: A Generalized Dose Optimization Design for Drug Combination Trials Maximizing Survival Benefit
Source: Pharm Stat. 2025 Nov 12;24(6):e70049. doi: 10.1002/pst.70049 (PMC12606548; doi:10.1002/pst.70049)
Supplement: Supplementary file 1 — Figure S1: Sensitivity analysis of the Great Wall design to different follow up times. Figure S2: Sensitivity analysis of the Great Wall design to different values of γ in the candidate dose set. Figure S3: Sensitivity analysis of the Great Wall design to different utility scores. Table S1: Simulation results of the Great Wall and Great Wall‐M designs. Sel % is the percentage of selecting the dose combination as the ODC. No Sel % is the percentages of claiming no ODC existing. Pat % is the percentages of patients treated at the dose combination. Boldface and gray highlighter indicates values for the true ODC. Table S2: Simulation results of the Great Wall designs under different cohort sizes in stage I. Great Wall‐3 and Great Wall‐6 use cohort sizes of 3 and 6 in stage I respectively. Sel % is the percentage of selecting the dose combination as the ODC. No Sel % is the percentages of claiming no ODC existing. Pat % is the percentages of patients treated at the dose combination. Boldface and gray highlighter indicates values for the true ODC. Table S3: Simulation results of the Great Wall design and modified Waterfall design. Sel % is the percentage of selecting the dose combination as the ODC. No Sel % is the percentages of claiming no ODC existing. Pat % is the percentages of patients treated at the dose combination. Boldface and gray highlighter indicates values for the true ODC. Table S4: Patient level data for toxicity and early efficacy outcomes for the trial example. Table S5: Patient level data for survival outcomes (in days). [file PST-24-0-s001.docx]

**Supplementary Materials for “Great Wall: A Generalized Dose Optimization Design for Drug Combination Trials Maximizing Survival Benefit” by Y. Han, Y. Qiu, Y. Zhao, I. Wan, L. Li, S. Liu and Y. Zang.**

***Figure S1. Sensitivity analysis of the Great Wall design to different follow up times.***


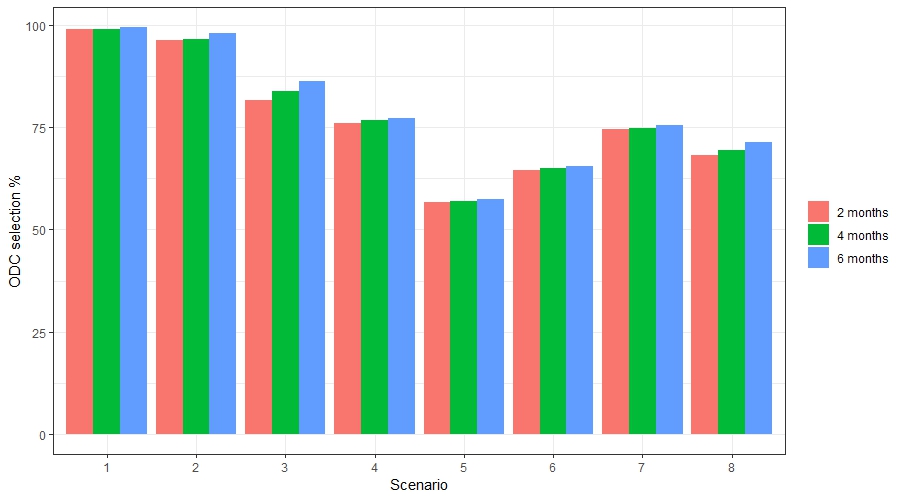


***Figure S2. Sensitivity analysis of the Great Wall design to different values of*** $\boldsymbol{\gamma}$ ***in the candidate dose set.***

**
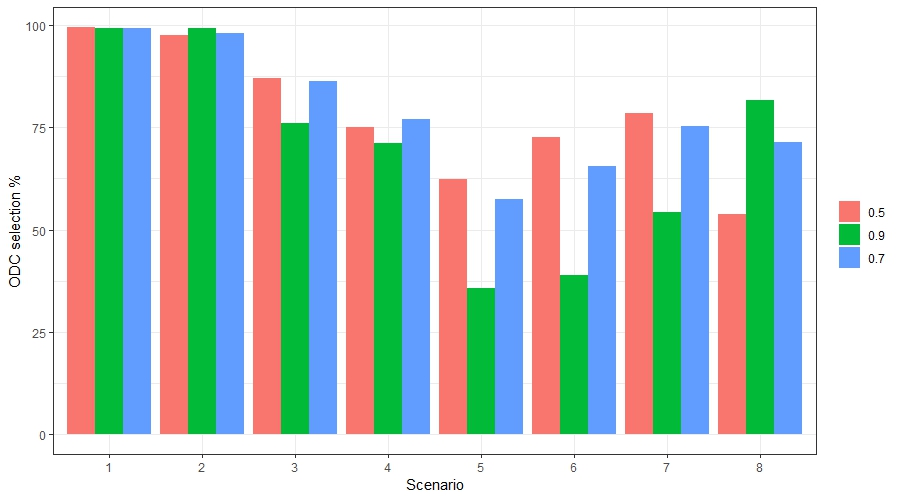
**

***Figure S3. Sensitivity analysis of the Great Wall design to different utility scores.***

***
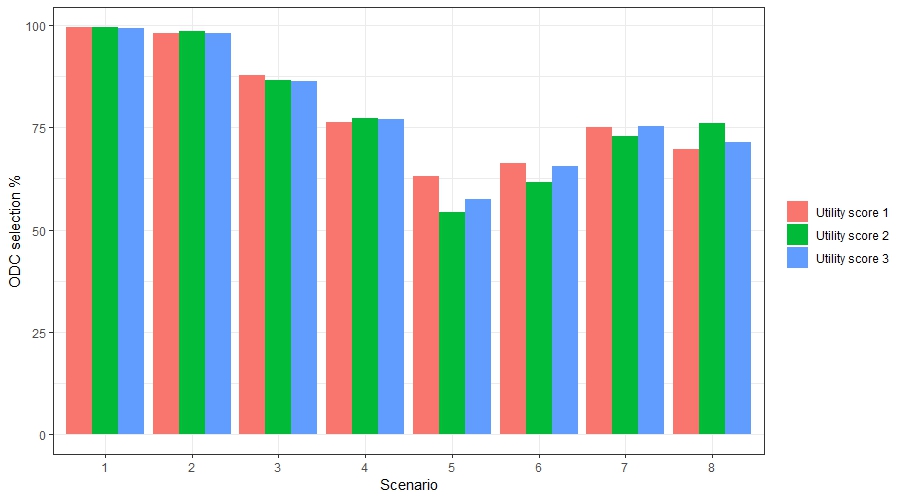
***

***Table S1. Simulation results of the Great Wall and Great Wall-M designs. Sel % is the percentage of selecting the dose combination as the ODC. No Sel % is the percentages of claiming no ODC existing. Pat % is the percentages of patients treated at the dose combination. Boldface*** ***and gray highlighter indicates values for the true ODC.***

| Designs | Sel % | | | No Sel % | Pat % | | | Sample size |
| --- | --- | --- | --- | --- | --- | --- | --- | --- |
| Scenario 1 | | | | | | | | |
| Great Wall | 0.0 | 0.0 | 0.0 | **99.4** | 14.3 | 3.2 | 0.5 | 32.2 |
|  | 0.6 | 0.0 | 0.0 |  | 65.3 | 13.7 | 3.0 |  |
| Great Wall-M | 0.0 | 0.0 | 0.0 | **97.3** | 15.4 | 3.3 | 0.6 | 38.3 |
|  | 2.7 | 0.0 | 0.0 |  | 64.2 | 13.6 | 3.0 |  |
| Scenario 2 | | | | | | | | |
| Great Wall | 0.2 | 0.3 | 0.0 | **98.0** | 23.5 | 10.9 | 5.1 | 72.0 |
|  | 0.0 | 0.3 | 1.2 |  | 32.6 | 19.3 | 8.6 |  |
| Great Wall-M | 0.4 | 0.4 | 0.0 | **97.3** | 22.6 | 13.0 | 6.1 | 72.0 |
|  | 0.0 | 0.3 | 1.6 |  | 29.7 | 18.6 | 10.0 |  |
| Scenario 3 | | | | | | | | |
| Great Wall | 5.6 | 0.1 | 0.0 | 3.1 | 25.1 | 11.2 | 5.3 | 71.7 |
|  | **86.3** | 4.9 | 0.0 |  | 31.3 | 18.7 | 8.4 |  |
| Great Wall-M | 8.8 | 0.3 | 0.2 | 3.3 | 23.5 | 13.8 | 6.5 | 71.9 |
|  | **82.9** | 4.6 | 0.1 |  | 28.6 | 17.9 | 9.7 |  |
| Scenario 4 | | | | | | | | |
| Great Wall | 5.3 | **77.1** | 0.3 | 1.9 | 19.3 | 21.6 | 8.9 | 73.1 |
|  | 0.3 | 11.1 | 4.0 |  | 18.0 | 20.6 | 11.6 |  |
| Great Wall-M | 5.8 | **74.7** | 0.8 | 1.3 | 19.9 | 17.8 | 10.7 | 73.5 |
|  | 0.3 | 10.3 | 6.7 |  | 19.8 | 19.0 | 12.8 |  |
| Scenario 5 | | | | | | | | |
| Great Wall | **57.4** | 8.6 | 0.8 | 19.1 | 21.9 | 20.2 | 8.2 | 71.2 |
|  | 1.7 | 6.3 | 6.0 |  | 19.5 | 19.3 | 11.0 |  |
| Great Wall-M | **57.0** | 8.7 | 1.5 | 8.9 | 19.8 | 16.7 | 9.9 | 72.0 |
|  | 3.5 | 6.7 | 13.7 |  | 21.2 | 19.5 | 12.9 |  |
| Scenario 6 | | | | | | | | |
| Great Wall | 20.4 | 8.9 | 0.1 | 2.5 | 24.1 | 17.0 | 7.6 | 71.8 |
|  | 1.2 | **65.6** | 1.2 |  | 19.6 | 20.7 | 11.0 |  |
| Great Wall-M | 20.0 | 10.9 | 0.2 | 2.6 | 21.3 | 16.9 | 9.2 | 72.6 |
|  | 3.0 | **62.2** | 1.1 |  | 21.6 | 19.0 | 12.0 |  |
| Scenario 7 | | | | | | | | |
| Great Wall | 0.3 | 11.2 | 8.4 | 3.8 | 14.3 | 18.7 | 16.7 | 72.1 |
|  | 0.1 | 0.7 | **75.4** |  | 14.6 | 16.1 | 19.5 |  |
| Great Wall-M | 0.6 | 10.5 | 11.7 | 3.2 | 18.1 | 16.5 | 14.2 | 72.6 |
|  | 1.0 | 1.4 | **71.6** |  | 18.2 | 17.1 | 16.0 |  |
| Scenario 8 | | | | | | | | |
| Great Wall | 0.3 | 4.2 | **71.4** | 0.5 | 15.0 | 18.2 | 22.7 | 73.8 |
|  | 0.0 | 1.3 | **22.3** |  | 13.7 | 15.0 | 15.5 |  |
| Great Wall-M | 0.3 | 3.9 | **51.7** | 0.2 | 17.9 | 17.2 | 15.2 | 73.9 |
|  | 0.2 | 1.9 | **41.9** |  | 16.8 | 16.7 | 16.2 |  |

***Table S2. Simulation results of the Great Wall designs under different cohort sizes in stage I. Great Wall-3 and Great Wall-6 use cohort sizes of 3 and 6 in stage I respectively. Sel % is the percentage of selecting the dose combination as the ODC. No Sel % is the percentages of claiming no ODC existing. Pat % is the percentages of patients treated at the dose combination. Boldface and gray highlighter indicates values for the true ODC.***

| Designs | Sel % | | | No Sel % | Pat % | | | Sample size |
| --- | --- | --- | --- | --- | --- | --- | --- | --- |
| Scenario 1 | | | | | | | | |
| Great Wall-3 | 0.0 | 0.0 | 0.0 | **99.4** | 14.3 | 3.2 | 0.5 | 32.2 |
|  | 0.6 | 0.0 | 0.0 |  | 65.3 | 13.7 | 3.0 |  |
| Great Wall-6 | 0.0 | 0.0 | 0.0 | **99.6** | 12.6 | 1.8 | 0.2 | 24.6 |
|  | 0.4 | 0.0 | 0.0 |  | 75.4 | 10.1 | 0.0 |  |
| Scenario 2 | | | | | | | | |
| Great Wall-3 | 0.2 | 0.3 | 0.0 | **98.0** | 23.5 | 10.9 | 5.1 | 72.0 |
|  | 0.0 | 0.3 | 1.2 |  | 32.6 | 19.3 | 8.6 |  |
| Great Wall-6 | 0.1 | 0.8 | 0.1 | **98.9** | 31.7 | 14.7 | 5.2 | 73.1 |
|  | 0.0 | 0.1 | 0.0 |  | 42.6 | 5.7 | 0.0 |  |
| Scenario 3 | | | | | | | | |
| Great Wall-3 | 5.6 | 0.1 | 0.0 | 3.1 | 25.1 | 11.2 | 5.3 | 71.7 |
|  | **86.3** | 4.9 | 0.0 |  | 31.3 | 18.7 | 8.4 |  |
| Great Wall-6 | 5.5 | 0.2 | 0.1 | 1.9 | 34.4 | 15.1 | 5.8 | 72.9 |
|  | **91.9** | 0.4 | 0.0 |  | 41.4 | 3.3 | 0.0 |  |
| Scenario 4 | | | | | | | | |
| Great Wall-3 | 5.3 | **77.1** | 0.3 | 1.9 | 19.3 | 21.6 | 8.9 | 73.1 |
|  | 0.3 | 11.1 | 4.0 |  | 18.0 | 20.6 | 11.6 |  |
| Great Wall-6 | 2.1 | **94.3** | 1.0 | 1.2 | 26.9 | 34.5 | 13.9 | 73.9 |
|  | 0.1 | 1.3 | 0.0 |  | 23.9 | 0.9 | 0.0 |  |
| Scenario 5 | | | | | | | | |
| Great Wall-3 | **57.4** | 8.6 | 0.8 | 19.1 | 21.9 | 20.2 | 8.2 | 71.2 |
|  | 1.7 | 6.3 | 6.0 |  | 19.5 | 19.3 | 11.0 |  |
| Great Wall-6 | **64.7** | 9.4 | 2.8 | 21.6 | 30.4 | 30.6 | 11.6 | 72.8 |
|  | 1.0 | 0.5 | 0.0 |  | 25.4 | 2.1 | 0.0 |  |
| Scenario 6 | | | | | | | | |
| Great Wall-3 | 20.4 | 8.9 | 0.1 | 2.5 | 24.1 | 17.0 | 7.6 | 71.8 |
|  | 1.2 | **65.6** | 1.2 |  | 19.6 | 20.7 | 11.0 |  |
| Great Wall-6 | 56.9 | 34.2 | 0.9 | 3.1 | 35.4 | 26.8 | 10.6 | 73.5 |
|  | 3.4 | **1.5** | 0.0 |  | 26.6 | 0.8 | 0.0 |  |
| Scenario 7 | | | | | | | | |
| Great Wall-3 | 0.3 | 11.2 | 8.4 | 3.8 | 14.3 | 18.7 | 16.7 | 72.1 |
|  | 0.1 | 0.7 | **75.4** |  | 14.6 | 16.1 | 19.5 |  |
| Great Wall-6 | 0.6 | 47.7 | 42.5 | 8.2 | 21.4 | 30.6 | 26.5 | 73.4 |
|  | 0.4 | 0.2 | **0.3** |  | 20.1 | 1.3 | 0.0 |  |
| Scenario 8 | | | | | | | | |
| Great Wall-3 | 0.3 | 4.2 | **71.4** | 0.5 | 15.0 | 18.2 | 22.7 | 73.8 |
|  | 0.0 | 1.3 | **22.3** |  | 13.7 | 15.0 | 15.5 |  |
| Great Wall-6 | 0.3 | 5.3 | **93.6** | 0.7 | 21.0 | 26.3 | 34.2 | 73.9 |
|  | 0.1 | 0.2 | **0.0** |  | 18.4 | 0.2 | 0.0 |  |

***Table S3. Simulation results of the Great Wall design and modified Waterfall design. Sel % is the percentage of selecting the dose combination as the ODC. No Sel % is the percentages of claiming no ODC existing. Pat % is the percentages of patients treated at the dose combination. Boldface and gray highlighter indicates values for the true ODC.***

| Designs | Sel % | | | No Sel % | Pat % | | | Sample size |
| --- | --- | --- | --- | --- | --- | --- | --- | --- |
| Scenario 1 | | | | | | | | |
| Great Wall | 0.0 | 0.0 | 0.0 | **99.4** | 14.3 | 3.2 | 0.5 | 32.2 |
|  | 0.6 | 0.0 | 0.0 |  | 65.3 | 13.7 | 3.0 |  |
| Modified  Waterfall | 0.3 | 0.2 | 0.0 | **97.8** | 12.2 | 2.0 | 1.1 | 30.1 |
|  | 0.2 | 0.5 | 1.0 |  | 79.2 | 3.8 | 1.7 |  |
| Scenario 2 | | | | | | | | |
| Great Wall | 0.2 | 0.3 | 0.0 | **98.0** | 23.5 | 10.9 | 5.1 | 72.0 |
|  | 0.0 | 0.3 | 1.2 |  | 32.6 | 19.3 | 8.6 |  |
| Modified  Waterfall | 0.3 | 1.0 | 0.7 | **91.8** | 26.8 | 11.5 | 2.2 | 71.6 |
|  | 0.0 | 0.0 | 6.2 |  | 33.9 | 17.1 | 8.6 |  |
| Scenario 3 | | | | | | | | |
| Great Wall | 5.6 | 0.1 | 0.0 | 3.1 | 25.1 | 11.2 | 5.3 | 71.7 |
|  | **86.3** | 4.9 | 0.0 |  | 31.3 | 18.7 | 8.4 |  |
| Modified  Waterfall | 14.1 | 2.4 | 1.7 | 11.8 | 29.3 | 13.4 | 2.4 | 71.9 |
|  | **63.6** | 6.2 | 0.2 |  | 30.7 | 15.7 | 8.6 |  |
| Scenario 4 | | | | | | | | |
| Great Wall | 5.3 | **77.1** | 0.3 | 1.9 | 19.3 | 21.6 | 8.9 | 73.1 |
|  | 0.3 | 11.1 | 4.0 |  | 18.0 | 20.6 | 11.6 |  |
| Modified  Waterfall | 9.7 | **69.4** | 0.4 | 4.6 | 21.2 | 30.8 | 6.4 | 73.9 |
|  | 0.4 | 10.4 | 5.1 |  | 15.7 | 12.5 | 13.3 |  |
| Scenario 5 | | | | | | | | |
| Great Wall | **57.4** | 8.6 | 0.8 | 19.1 | 21.9 | 20.2 | 8.2 | 71.2 |
|  | 1.7 | 6.3 | 6.0 |  | 19.5 | 19.3 | 11.0 |  |
| Modified  Waterfall | **45.3** | 7.5 | 1.4 | 27.7 | 22.5 | 25.9 | 5.1 | 72.5 |
|  | 1.5 | 5.4 | 11.2 |  | 19.2 | 14.2 | 13.1 |  |
| Scenario 6 | | | | | | | | |
| Great Wall | 20.4 | 8.9 | 0.1 | 2.5 | 24.1 | 17.0 | 7.6 | 71.8 |
|  | 1.2 | **65.6** | 1.2 |  | 19.6 | 20.7 | 11.0 |  |
| Modified  Waterfall | 26.2 | 24.0 | 0.3 | 5.4 | 25.2 | 25.2 | 4.5 | 73.4 |
|  | 3.9 | **36.6** | 3.6 |  | 19.1 | 14.4 | 11.6 |  |
| Scenario 7 | | | | | | | | |
| Great Wall | 0.3 | 11.2 | 8.4 | 3.8 | 14.3 | 18.7 | 16.7 | 72.1 |
|  | 0.1 | 0.7 | **75.4** |  | 14.6 | 16.1 | 19.5 |  |
| Modified  Waterfall | 1.0 | 21.9 | 7.9 | 9.4 | 17.5 | 24.2 | 13.8 | 73.4 |
|  | 1.1 | 1.0 | **57.7** |  | 13.7 | 5.4 | 25.4 |  |
| Scenario 8 | | | | | | | | |
| Great Wall | 0.3 | 4.2 | **71.4** | 0.5 | 15.0 | 18.2 | 22.7 | 73.8 |
|  | 0.0 | 1.3 | **22.3** |  | 13.7 | 15.0 | 15.5 |  |
| Modified  Waterfall | 1.3 | 15.6 | **31.6** | 3.1 | 15.7 | 25.6 | 19.4 | 73.9 |
|  | 0.5 | 1.2 | **46.7** |  | 10.9 | 1.5 | 26.9 |  |

**Trial illustration**

In this section, we provide an illustrative example using the same setting of scenario 3 from Table 1 and set $\emptyset_{T}=0.3$, $\emptyset_{E}=0.25$ and $\emptyset_{S}=0.3$, with the only difference being that $n_{1}=18$, $n_{2}=18$ and $n_{3}=18$. The detailed patient level data are expressed in Tables S4 and S5. The trial starts at the first sub-path $\mathcal{S}_{1}=\{A_{1}B_{1}, A_{2}B_{1}, A_{2}B_{2}, A_{2}B_{3}\}$. We keep escalating the dose combination until identifying $A_{2}B_{2}$ as an overly toxic dose combination with $\hat{p_{T}}\left( A_{2}B_{2} \right)=\frac{2}{3}$, which is greater than the cut-off value $\psi=0.358$. So the first wall is built at $A_{2}B_{2}$ and we move forward to the second sub-path $\mathcal{S}_{2}=\{A_{1}B_{2}, A_{1}B_{3}\}$. By escalating the dose within $\mathcal{S}_{2}$ we identify $A_{1}B_{3}$ as overly toxic with $\hat{p_{T}}\left( A_{1}B_{3} \right)=\frac{2}{3}$, and build the second wall at $A_{1}B_{3}$. Hence, we identify the admissible set of toxicity at stage 1 as ${\mathcal{A}_{T,1}=\{A}_{1}B_{1}, A_{2}B_{1},A_{1}B_{2}\}$.

Based on the early-efficacy outcome, we calculate the posterior probabilities as $\Pr\left( p_{E}\left( A_{1}B_{1} \right)>0.25 | \mathcal{D}_{1} \right)=0.949$, $\Pr\left( p_{E}\left( A_{2}B_{1} \right)>0.25 | \mathcal{D}_{1} \right)=0.949$, and $\Pr\left( p_{E}\left( A_{1}B_{2} \right)>0.25 | \mathcal{D}_{1} \right)=0.996$, which are all greater than the cut-off value 0.05. So we identity the admissible set ${\mathcal{A}_{1}=\{A}_{1}B_{1}, A_{2}B_{1},A_{1}B_{2}\}$ and move to stage 2.

It is worth noticing that although the maximum sample size is set at 18, we only use 15 of them in stage 1. Therefore, as a seamless design, we combine the remaining 3 patients into stage 2 and equally randomize 21 patients among $\mathcal{A}_{1}$. Then, we apply the matrix isotonic regression to the accumulated toxicity data and get the DLT estimates (0, 0.2, 0.572, 0.1, 0.572) for all the tried dose combinations ($A_{1}B_{1}, A_{2}B_{1}, A_{2}B_{2}, A_{1}B_{2}, A_{1}B_{3}$). The MTD contour is identified as $A_{2}B_{1}$ and $A_{1}B_{2}$ and consequently ${\mathcal{A}_{T,2}=\{A}_{1}B_{1}, A_{2}B_{1},A_{1}B_{2}\}$. Again, all the dose combinations in $\mathcal{A}_{T,2}$ report acceptable early-efficacy profiles. In terms of utility values, $A_{1}B_{2}$ yields the highest mean utility of 0.84, and the values for other two dose combinations are 0.70 and 0.68, which are both greater than the cut-off values of $0.84\times0.7=0.588$. Therefore, we identify the candidate dose set as $\mathcal{C}_{2}{=\{A}_{1}B_{1}, A_{2}B_{1},A_{1}B_{2}\}$ and move to stage 3.

In stage 3, we continue to equally randomize the last 18 patients among $\mathcal{C}_{2}$ and collect the toxicity and PFS outcomes for all the patients in the trial. Based on the updated toxicity data we reinsure that the final MTD contour is still identified at $A_{2}B_{1}$ and $A_{1}B_{2}$ so the candidate dose set keep unchanged. The estimated survival rates are 0.50, 0.375, and 0.312 for $A_{1}B_{1}$, $A_{2}B_{1}$ and $A_{1}B_{2}$, respectively. Therefore, the final ODC is selected as $A_{1}B_{1}$, and a Go decision is made because 0.5>0.3.

***Table S4. Patient level data for toxicity and early efficacy outcomes for the trial example.***

|  | Cohort | Dose  Combination | Current # $(Y_{T},Y_{E})$ | | | | Accumulated # $(Y_{T},Y_{E})$ | | | |
| --- | --- | --- | --- | --- | --- | --- | --- | --- | --- | --- |
|  |  |  | (0, 0) | (0, 1) | (1, 0) | (1, 1) | (0, 0) | (0, 1) | (1, 0) | (1, 1) |
| Stage 1 | 1 | $A_{1}B_{1}$ | 1 | 2 | 0 | 0 | 1 | 2 | 0 | 0 |
|  | 2 | $A_{2}B_{1}$ | 1 | 1 | 0 | 1 | 1 | 1 | 0 | 1 |
|  | 3 | $A_{2}B_{2}$ | 1 | 0 | 1 | 1 | 1 | 0 | 1 | 1 |
|  | 4 | $A_{1}B_{2}$ | 0 | 3 | 0 | 0 | 0 | 3 | 0 | 0 |
|  | 5 | $A_{1}B_{3}$ | 1 | 0 | 1 | 1 | 1 | 0 | 1 | 1 |
| Stage 2 | 6 | $A_{1}B_{1}$ | 4 | 3 | 0 | 0 | 5 | 5 | 0 | 0 |
|  | 7 | $A_{2}B_{1}$ | 3 | 3 | 0 | 1 | 4 | 4 | 0 | 2 |
|  | 8 | $A_{1}B_{2}$ | 2 | 4 | 0 | 1 | 2 | 7 | 0 | 1 |
| Stage 3 | 9 | $A_{1}B_{1}$ | 2 | 3 | 0 | 1 | 7 | 8 | 0 | 1 |
|  | 10 | $A_{2}B_{1}$ | 1 | 4 | 0 | 1 | 5 | 8 | 0 | 3 |
|  | 11 | $A_{1}B_{2}$ | 2 | 2 | 2 | 0 | 4 | 9 | 2 | 1 |

***Table S5. Patient level data for survival outcomes (in days)***

| $A_{2}B_{1}$ | $A_{1}B_{1}$ | $A_{1}B_{2}$ | $A_{2}B_{2}$ | $A_{1}B_{3}$ |
| --- | --- | --- | --- | --- |
| 131 | 44 | 82 | 113 | 180 |
| 180 | 180 | 110 | 156 | 53 |
| 137 | 180 | 180 | 118 | 180 |
| 40 | 180 | 149 |  |  |
| 178 | 128 | 180 |  |  |
| 161 | 95 | 77 |  |  |
| 180 | 180 | 141 |  |  |
| 150 | 92 | 75 |  |  |
| 180 | 9 | 180 |  |  |
| 180 | 180 | 132 |  |  |
| 59 | 180 | 180 |  |  |
| 180 | 180 | 114 |  |  |
| 83 | 87 | 180 |  |  |
| 147 | 180 | 119 |  |  |
| 95 | 136 | 71 |  |  |
| 180 | 148 | 40 |  |  |
